# Association of Per- and Polyfluoroalkyl Substances with Pan-Cancers Associated with Sex Hormones

**DOI:** 10.3390/toxics13060501

**Published:** 2025-06-14

**Authors:** Elizabeth Olarewaju, Emmanuel Obeng-Gyasi

**Affiliations:** 1 Department of Built Environment, North Carolina A&T State University, Greensboro, NC 27411, USA; 2 Environmental Health and Disease Laboratory, North Carolina A&T State University, Greensboro, NC 27411, USA

**Keywords:** chemical mixtures, cancer biomarkers, NHANES, Bayesian kernel machine regression, environmental exposure

## Abstract

Per- and polyfluoroalkyl substances (PFASs) are ubiquitous environmental contaminants with potential endocrine-disrupting properties. This study examines the association between exposure to multiple PFASs and pan-cancers associated with sex hormones (PCSH) while accounting for potential non-linear relationships and interactions. We analyzed data from the National Health and Nutrition Examination Survey (NHANES), spanning two-year cycles from 1999 to 2012 and including 14,373 participants. Serum concentrations of six PFAS—perfluorooctanoic acid (PFOA), perfluorooctanesulfonic acid (PFOS), perfluorohexanesulfonic acid (PFHxS), perfluorodecanoic acid (PFDE), perfluorononanoic acid (PFNA), and perfluoroundecanoic acid (PFUA)—were assessed for their relationship with PCSH. The statistical analyses included descriptive statistics, Spearman and Pearson correlation analyses, and both linear and logistic regression models. Additionally, Bayesian kernel machine regression (BKMR) was applied to capture potential nonlinear relationships and interactions. The initial *t*-tests showed a statistically significant difference in PFOS levels between individuals with and without PCSH (*p* = 0.0022), with higher mean PFOS levels in the PCSH group. Chi-square tests revealed a significant association between ethnicity and PCSH (*p* < 0.001). Linear and logistic regression analyses revealed significant associations for PFOS. BKMR analysis identified PFOA as having the highest posterior inclusion probability, indicating its importance in explaining PCSH risk. Univariate exposure-response analysis revealed limited individual PFAS effects. However, bivariate analysis indicated a complex U-shaped interaction pattern among many joint PFAS assessments. The overall exposure effect analysis suggested that the combined impact of all PFASs was more strongly associated with PCSH at exposure levels below the 0.5 quantile compared to higher levels. Single-variable interaction analyses highlighted PFOA and PFOS as the most interactive PFASs when evaluating their interaction with combined exposure to all other PFASs. In summary, while the initial findings suggested a positive association between PFOS and PCSH, the BKMR analysis revealed complex non-linear relationships and interactions among PFAS. These findings highlight the importance of evaluating PFASs as a mixture rather than as individual chemicals and using techniques that can capture non-linear relationships and interactions.

## 1. Introduction

Per- and polyfluoroalkyl substances (PFASs) are a group of man-made chemicals that have been widely used in various industrial and consumer products [1], including paper and food packaging, leather and apparel, carpet, coatings, textiles, rubber, oil-repelling containers, semiconductors, and plastics, due to their water- and grease-resistant properties [2,3]. These chemicals are of significant concern, due to their prolonged persistence in the environment and their detrimental impacts on human and environmental health, which are known or can be inferred from exposure to these compounds [4,5].

The Organization for Economic Co-operation and Development defines PFASs as “fluorinated substances that contain at least one fully fluorinated methyl or methylene carbon atom (without any H/Cl/Br/I atom attached to it), i.e., with a few noted exceptions, any chemical with at least a perfluorinated methyl group (–CF3) or a perfluorinated methylene group (–CF2–) is a PFAS” [6]. This definition enables consistency across compounds and facilitates the distinction between PFASs and non-PFASs, particularly for non-experts. PFASs, as a class of chemical substances, include up to 4000 different variants, of which 250 are being manufactured in quantities that have become significant globally [7].

One common characteristic of PFASs, which has become a significant health concern over the years, is that many of these compounds break down very slowly and can build up in people, animals, and the environment over time. The global distribution of PFASs is extensively driven by atmospheric transport; after deposition, dispersion within the ecosystem declines with increasing molecular mass, while biological accumulation increases [8]. Other sources of exposure to PFASs include food [9], drinking water, aquatic environments [10], manufacturing, landfills, waste streams, and treatment systems [11].

There is ongoing research into the association of PFAS exposure with various health conditions, including developmental effects, adverse reproductive health, and an increased risk of certain types of cancer. PFASs have been associated with elevated levels of preeclampsia, obesity in children, pregnancy-related diabetes, and the restriction of fetal development [12,13,14]. The relationship between PFAS exposure and cancer is a complex and evolving area of study, and researchers have been particularly interested in understanding any potential links between PFASs and hormone-related cancers. Hormone-related cancers include breast, ovarian, prostate, and endometrial cancers, among others, as they are influenced by sex hormones such as estrogen and testosterone. Although data from epidemiological studies are limited, PFASs are extremely persistent endocrine disruptors that may be a contributing factor in the development of breast cancer [15].

Perfluoro-octanoic acid (PFOA) and perfluoro-octane sulfonate (PFOS) are synthetic substances that are extensively utilized in manufacturing and consumer industries. Although there is an inadequate amount of data that characterize the impacts of all PFAS variants in the ecosystems, PFOA and PFOS are widely known and account for 21% and 39% of the ECOTOX Knowledgebase, respectively [16]. These compounds are classified as endocrine disruptors and potential carcinogens, with a positive association between serum PFOS levels and hormone receptor-positive breast tumors [15]. Some of these chemicals have been in commercial use since the 1940s, with usage common in non-stick and stain-resistant consumer products, food packaging, fire-fighting foam, and industrial processes. Perfluorooctanoic acid (PFOA) or C8 is an emulsifier and surfactant that is widely utilized in the manufacturing process of polymers such as Teflon and Gore-Tex. It is found in relatively small but quantifiable amounts in the serum of a significant proportion of people living in the United States, with a median ≈ of 4 µg/L [17]. PFOA and PFOS have been identified as possessing estrogen-mimicking characteristics, which can inhibit the pathways of biological metabolism that are regulated by these hormones and can also bind to sex hormone-binding globulin (SHBG)—a protein that has an essential role in transporting and regulating the synthesis of sex hormones in the bloodstream [18]. Their binding to SHBG can interfere with the synthesis and distribution of sex hormones in the human body while altering the physiological functions of the reproductive system. PFOS and PFOA are no longer manufactured in the United States. However, they are still being produced internationally for use in consumer goods and are imported into the United States. These consumer goods include paper and packaging, leather and apparel, carpet, coatings, textiles, rubber, and plastics.

Globally, regulatory agencies have increasingly recognized the health and environmental risks posed by PFASs. The U.S. Environmental Protection Agency (EPA) has recently established a maximum contaminant level (MCL) of 4 parts per trillion (ppt) for perfluorooctanoic acid (PFOA) and perfluorooctanesulfonic acid (PFOS) in drinking water, along with proposed limits for other PFASs such as PFNA, PFHxS, and HFPO-DA (GenX), through a cumulative hazard index approach [19]. Similarly, the European Union has taken action through the registration, evaluation, authorization, and restriction of chemicals (REACH) framework, with certain PFASs (including PFOA and PFOS) listed as substances of very high concern (SVHCs) [20], and with broader restrictions proposed under the Stockholm Convention on Persistent Organic Pollutants. Despite these efforts, permissible exposure levels and regulatory enforcement vary significantly across countries, reflecting the challenges in developing unified global standards and countermeasures on human exposure to PFASs [21]. Understanding PFAS exposure in the context of these evolving guidelines underscores the need to research the varying health implications in order to inform regulatory decision-making and public health strategies.

Sex hormones are a group of hormones produced by the endocrine cells that play pivotal roles in the development and regulation of sexual characteristics and the functionalities of tissues associated with the reproductive system. Primary sex hormones have been identified as estrogen in females, which is responsible for the regulation and growth of the female reproductive system and secondary sexual features, and testosterone in males, which also has properties that are used medically to stimulate tissue growth [18,22]; these hormones comprise total testosterone (TT), estradiol (E_2_), sex hormone-binding globulin (SHBG), progesterone (P), and prolactin (PRL) [23,24]. The regular synthesis of these hormones regulates human reproductive health, and in situations of imbalance, various diseases, including certain types of cancer, are likely to occur. Cancers associated with sex-hormone variations and imbalances include prostate, testis, breast, and ovarian cancers. Studies have shown that there are potentially increasing risks of ovarian and breast cancer with elevated estradiol and estrogen levels. In contrast, testis and prostate cancers are associated with testosterone and estradiol [25].

Previous studies have examined the effects of sex hormones and prostate cancer development, heavy metals, and the progression of breast cancer [26,27]. Multiple epidemiologic studies have been carried out that link PFAS exposure to cancer and cancers associated with sex hormones, although the conclusions are not definitive [28,29,30,31,32,33]. This study sought to examine the effects of PFASs on pan-cancers associated with sex hormones. The hypothesis of this study posited that exposure to PFASs has a significant impact on the development of cancers that are strongly associated with sex hormones.

## 2. Materials and Methods

### 2.1. Study Population

The study utilized data from the National Health and Nutrition Examination Survey (NHANES), a comprehensive cross-sectional survey designed to assess the health and nutritional status of the United States population. The NHANES was conducted by the National Center for Health Statistics (NCHS). The data contained within NHANES was gathered using questionnaires; in addition, the team also conducted analyses on blood and urine samples obtained from participants who provided informed consent.

For this study, the dataset encompassed information grouped in 2-year cycles from 1999 to 2012. It specifically included adults aged over 20 years who met the inclusion criteria of everybody who had complete data for the PFASs of interest. In total, the study population consisted of 14,373 individuals. Among these, there were 14,087 individuals with no history of cancer, with 282 having pan-cancers associated with sex hormones.

### 2.2. Cancers Linked to Sex Hormones Across Sexes

In the human body, sex hormones are primarily secreted by the ovaries and testes. These hormones play a significant role in influencing the development of the breast and prostate glands. Throughout the process of cancer development, the levels of sex hormones have a notable impact on the occurrence of ovarian and testicular cancers. Furthermore, these hormone levels can also affect the development of breast and prostate cancers.

In our study, we encompass these four specific types of cancer and collectively refer to them as “pan-cancers associated with sex hormones.” To determine if individuals were affected by cancers related to sex hormones, we utilized specific questions related to each type of cancer. Respondents were asked about their history of breast cancer, testicular cancer, ovarian cancer, and prostate cancer through the following questions:

- For breast cancer: “Have you ever been informed that you have had breast cancer?” (yes/no)
- For testicular cancer: “Have you ever been told that you had testicular cancer?” (yes/no)
- For ovarian cancer: “Have you ever been informed that you had ovarian cancer?” (yes/no)
- For prostate cancer: “Have you ever been told that you had prostatic cancer?” (yes/no)

This approach allowed us to assess the presence of sex hormone-related cancers in the surveyed individuals across both genders.

### 2.3. Assessment of PFAS Exposure

PFAS quantification in serum samples was conducted by the National Center for Environmental Health (NCEH) at the Centers for Disease Control and Prevention (CDC) using on-line solid-phase extraction coupled to high-performance liquid chromatography and tandem mass spectrometry (SPE-HPLC-TIS-MS/MS) [34,35]. The analytical system included a Symbiosis online SPE-HPLC system (Spark Holland Inc., Plainsboro, NJ, USA) coupled to a triple quadrupole mass spectrometer with a TurboIonSpray ionization source (SCIEX, Foster City, CA, USA). Serum samples (50 µL) were diluted with formic acid (EM Science, Gibbstown, NJ, USA) and injected into the column-switching system, which concentrated the analytes and separated them chromatographically. Quantification was based on isotope dilution using native and mass-labeled PFAS standards (Wellington Laboratories, Guelph, ON, Canada). Quality control procedures included calibration standards, reagent blanks prepared with PFAS-free calf serum (Gibco, Thermo Fisher Scientific, Grand Island, NY, USA), and matrix spike recoveries. Lower limits of detection were established at 0.10 ng/mL for each analyte. In this study, we focused on six PFAS measured across NHANES 1999–2012: perfluorooctanoate (PFOA), perfluorooctane sulfonate (PFOS), perfluorohexane sulfonate (PFHxS), perfluorodecanoate (PFDeA), perfluorononanoate (PFNA), and perfluoroundecanoate (PFUA). Detailed protocols are available in the CDC NHANES laboratory documentation.

### 2.4. Statistical Analysis

#### 2.4.1. Descriptive Statistics

Descriptive analyses were conducted to summarize the demographic characteristics, exposure levels, and prevalence of sex hormone-related cancers among the study population. Continuous variables, such as age and serum PFAS concentrations, were presented as means with standard deviations or medians with interquartile ranges, depending on their distribution. Categorical variables, such as sex and cancer history, were presented as frequencies and percentages. Differences between groups (e.g., individuals with and without sex hormone-related cancers) were assessed using independent *t*-tests for continuous variables and chi-square tests for categorical variables.

#### 2.4.2. Correlation Analysis

To explore the significance and direction of associations between PFAS concentrations and pan-cancers associated with sex hormones (PCSH), both Spearman and biserial Pearson correlations were utilized. Assuming a normal distribution, the Pearson correlation assesses the linear relationships between exposure and outcome variables, while the Spearman correlation captures monotonic but potentially non-linear relationships, offering robustness to non-normally distributed data.

The Pearson correlation coefficient (r) equation is described as follows:

$$r=\frac{\sum \left(X_{i}-\bar{x}\right)\left(Y_{i}-\bar{Y}\right)}{\sqrt{\sum {\left(X_{i}-\bar{x}\right)}^{2}\sum {\left(Y_{i}-\bar{Y}\right)}^{2}}}$$

where X_i_ represents individual PFAS concentrations; $\bar{x}$ indicates the mean of all PFAS concentrations; Y_i_ represents individual cancer outcomes; $\bar{Y}$ is the mean of all cancer outcomes; and Σ indicates the summation of all data points.

This coefficient ranges from −1 to +1, where values close to +1 indicate a strong positive correlation, values close to −1 indicate a strong negative correlation, and values near 0 indicate little to no linear correlation.

The Spearman rank correlation can be expressed mathematically, as follows:

$$r_{s}=1-\frac{6\sum d_{i}^{2}}{n(n^{2}-1)}$$

In this context, $d_{i}$ represents the difference between the ranks of the i-th pair and n denotes the total number of observations in the dataset.

The Spearman correlation coefficient, $r_{s}$, ranges from −1 to +1. A positive = $r_{s}$ value suggests a direct (positive) relationship between the two variables, while a negative value implies an inverse (negative) relationship. An $r_{s}$ of zero indicates that there is no monotonic association between the variables.

#### 2.4.3. Logistic Regression

To evaluate the association between PFAS exposure and the odds of sex hormone-related cancers, logistic regression models were employed.

The logistic regression equation used in the study is described below:

log(p/(1 − p)) = β_0_ + β_1_X_1_ + β_2_X_2_ + ... + β_n_X_n_

where p is the probability of developing cancer associated with sex hormones; p/(1 − p) is the odds of developing cancer; log(p/(1 − p)) is the log odds; β_0_ represents the intercept; β_1_, β_2_, ..., β_n_ are the regression coefficients; and X_1_, X_2_, ..., X_n_ are the predictor variables.

Odds ratios (ORs) and 95% confidence intervals (CIs) were calculated for each PFAS compound. ORs > 1 indicated that higher PFAS exposure was associated with increased odds of developing cancers associated with sex hormones, while ORs < 1 suggested otherwise. Also, if the 95% CI included 1.0, the association was identified to be statistically insignificant at the *p* < 0.05 level, while a 95% CI without 1.0 implied a statistically significant association. Initial models included adjustments for age, sex, race/ethnicity, body mass index (BMI), smoking status, and socioeconomic status. Potential effect modification by sex was assessed by including the interaction terms between sex and PFAS concentrations in the models.

#### 2.4.4. Bayesian Kernel Machine Regression (BKMR)

BKMR was utilized to explore the potential non-linear and interactive effects of PFAS mixtures on sex hormone-related cancers. This approach enables the assessment of both individual and combined effects of multiple PFAS compounds while accounting for complex interactions. The use of linear and logistic regression techniques is based on the underlying premise that linear relationships exist between the dependent and independent variables of interest. However, these linear approaches may not sufficiently capture the complexities of exposures in the real world, where instances of non-linear interactions occur in multi-pollutant environmental settings. Other mixture methods, such as the weighted quantile sum, also assume linearity. By leveraging Bayesian modeling and flexible kernel functions, BKMR effectively captures complex dependencies and interactions among exposures. This approach uncovers patterns that other modeling techniques may overlook, leading to more accurate, robust, and insightful findings.

In this study, we employed BKMR alongside the Markov chain Monte Carlo sampling method, as outlined by Bobb et al. [36]. We performed 5000 iterations in the analysis and posterior inclusion probabilities (PIPs) were calculated to identify those PFAS compounds with the most significant contributions to cancer risk. Additionally, exposure-response functions were plotted to visualize the relationships between PFAS mixtures and cancer outcomes.

The modeling framework adopted for the BKMR analysis in this study is as follows:

Y_i_ = h(z_i_) + x_i_^T^β + Ɛ_i_     I = 1, …, n

where Y_i_ represents the health outcome (one or more cancers associated with sex hormones); z_i_ = (z_i1_, …, z_iM_)^T^ denotes a vector of exposure variables; h is a flexible function capturing the nonlinear and interactive effects of PFAS; x is a vector of covariates assumed to have a linear relationship with the outcome; and Ɛ_i~_N (0, σ^2^) indicates an error term.

Age, sex, ethnicity, and BMI were adjusted for as covariates in all models, while in non-Bayesian analysis, a *p*-value < 0.05 indicated a statistically significant association. Data analysis was conducted using R (version 4.2.3; R Foundation for Statistical Computing, Vienna, Austria).

## 3. Results

### 3.1. Descriptive Statistics of Critical Variables in the Study

The study (Table 1) comprised a total of 14,373 participants, with a relatively balanced gender distribution, where 49.52% identified as male and 50.48% as female. The racial and ethnic composition of the participants was notably diverse, with non-Hispanic Whites being the predominant group at 40.37%, non-Hispanic Blacks at 22.49%, Mexican Americans at 18.88%, other races, including multi-racial individuals, at 9.62%, and other Hispanic individuals at 8.63%. In addition, 1.96% of the people in the study were reported to have cancers related to sex hormones.

Table 2 presents the mean levels of critical variables in the study, with PFOS showing the highest mean concentration level and variability (13.99 ± 16.02), followed by PFOA (3.31 ± 2.89), while PFUA has the lowest mean levels (0.24 ± 0.46). PFOA and PFOS exhibit extreme maximum values (104 and 435) when compared to their mean levels, which may suggest high exposure scenarios. The variability in the mean levels indicates the presence of distinct exposure groups in the dataset, with significant variations in the observed PFAS levels. This suggests that exposure sources are diverse and unevenly distributed across different populations, making it challenging to characterize exposure levels. Consequently, environmental risk assessments must consider both populations with lower exposure levels and those with higher exposure concentrations. The average age and BMI of participants in the study were 42.69 and 28.15, respectively.

### 3.2. t-Tests and Chi-Square Tests

Table 3 presents the results of the *t*-tests comparing the mean levels of various PFAS variables between individuals with PCSH and those without PCSH. From the analysis, PFOS is observed to make a statistically significant difference between individuals with PCSH and those without, having a *p*-value of 0.0022. Individuals with PCSH have higher mean PFOS levels (17.23 ng/mL) compared to those without PCSH (13.93 ng/mL). This may suggest an association of PFOS levels with the presence of PCSH in the study population. For other PFAS concentrations—PFOA, PFHxS, PFDE, PFNA, and PFUA— there are no statistically significant differences between individuals with PCSH and those without PCSH groups, as *p*-values were > 0.05, highlighting no observable associations in the context of this analysis.

Table 4 indicates a higher proportion of males (53.55%) in the group of individuals with PCSH compared to other groups. Non-Hispanic Whites are more strongly represented in the group with PCSH (57.09%) compared to their proportion in the no-PCSH group (40.04%). Conversely, Mexican Americans are notably underrepresented in the PCSH group (6.03%), compared to their proportion in the no-PCSH group (19.14%). This suggests that being non-Hispanic White may be associated with a higher likelihood of having PCSH.

The chi-square tests to determine the differences between groups of individuals with PCSH and those without sex hormone-related cancers according to sex and ethnicity revealed a statistically significant association between ethnicity and PCSH, where χ^2^: 46.22 and the *p*-value < 0.0001. This suggests a strong relationship between ethnicity and the presence or absence of PCSH in the study.

### 3.3. Spearman and Pearson Correlation Analysis

**Figure 1:**
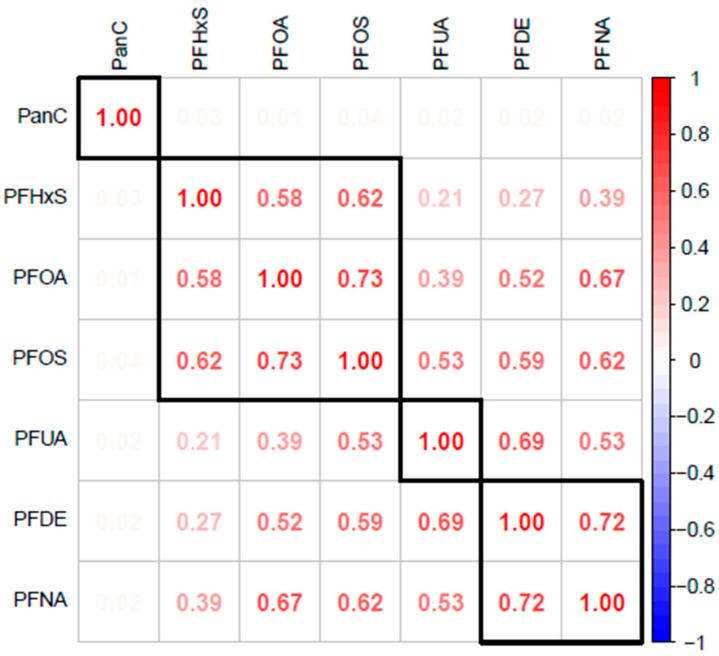
The Spearman correlation matrix reveals the relationships between various PFAS, including PFOA, PFOS, PFHxS, PFUA, PFDE, PFNA, and PCSH.

To explore the relationships between the variables, Spearman correlation analysis was performed using PFHxS, PFOA, PFOS, PFUA, PFDE, PFNA, and PCSH. The results from the matrix (Figure 1) indicate significant correlations between PFAS compounds. Strong correlations were observed between PFOS and PFOA (ϱ = 0.73) and between PFNA and PFDE (ϱ = 0.72), while there is a moderate correlation between PFOS and PFHxS (ϱ = 0.62) and between PFOA and PFHxS (ϱ = 0.58), which is a possible indication of an overlap in exposure pathways. Correlations between PFHxS and PFUA (ϱ = 0.21) and between PFDE and PFHxS (ϱ = 0.27) are considered weak or near-zero, suggesting that these compounds exhibit independent behaviors. For this analysis, correlations with *ϱ* values ≥ 0.7 were considered strong, while values in the range of 0.50–0.70 and those below 0.30 were considered moderate and weak, respectively.

**Figure 2:**
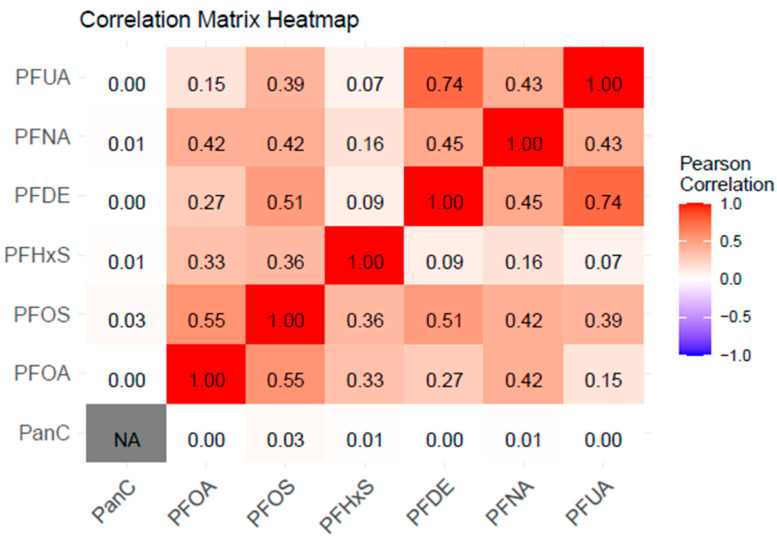
This image represents a correlation matrix heatmap showing correlations among the outcome and exposure variables. The values range from −1 (strong negative correlation, indicated by the blue color on the legend) to +1 (strong positive correlation, indicated by the red color), with 0 indicating no correlation (represented by the grey/white area).

Figure 2 displays a Pearson correlation matrix heatmap. The results reveal that PFUA and PFDE have the strongest correlation of 0.74, while PFOS and PFOA have a correlation of 0.55. PFUA, PFDE, and PFOA do not have any observable correlations with PCSH from the heatmap. The matrix indicates that while various PFAS compounds show moderate to strong correlations with each other (suggesting that they may co-occur in environments or have similar sources), there appears to be no significant correlation between any of these PFAS compounds and pan-cancers, based on this analysis. There are no negative correlations to be observed in the heatmap.

### 3.4. Regression Analysis

The linear regression analysis (Table 5) presents the estimates of the association between PCSH and its predictor PFAS variables, adjusted for sex, age, BMI, and ethnicity. From the table, PFOA (*β* = −0.067*, p* = 0.036) indicates a statistically significant inverse association with PCSH*,* which suggests that higher PFOA levels are associated with decreased odds of pan-cancers.

The logistic regression analysis (Table 6) shows PFOA having a statistically significant inverse association with the outcome variable, PCSH, with an odds ratio of 0.935 (95% CI: 0.876–0.994), implying that a unit increase in PFOA concentration will result in a decrease in the odds of PCSH risk. However, from this analysis, other PFAS variables—PFOS, PFHxS, PFDE, PFNA, and PFUA—do not show statistically significant associations with PCSH.

### 3.5. Bayesian Kernel Machine Regression

Given the statistically significant correlations and anticipated complex interactions within our dataset, we performed BKMR. Classical linear regression models, predicated on the linear relationships between variables, are often inadequate for capturing the non-linear and non-additive interactions prevalent among environmental pollutants. BKMR, leveraging Bayesian modeling and adjustable kernel functions, provides a more robust framework for analyzing these intricate relationships, leading to a more precise and comprehensive understanding of our data.

BKMR was performed to assess the non-linear relationships and interactions between PFAS variables and the outcome of this study. With BKMR, the posterior inclusion probability, univariate, bivariate, overall, and single-variable interactive exposure-response effects were evaluated.

#### 3.5.1. Posterior Inclusion Probability When Quantifying the Influence of PFAS Exposures in PCSH

The posterior inclusion probability (Table 7) was assessed to determine the variable that can be identified as the most relevant predictor for pan-cancers associated with sex hormones. The results from the analysis revealed PFOA and PFUA as the most relevant predictor variables, having the highest PIPs of 0.1792 and 0.1036, respectively.

#### 3.5.2. Univariate Analysis of the Isolated Effects of Each PFAS Variable on PCSH

**Figure 3:**
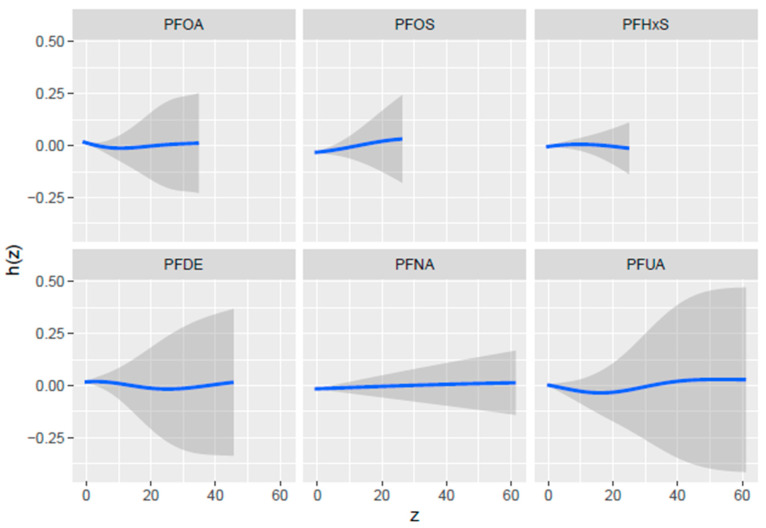
Univariate exposure-response functions with a 95% confidence interval for the relationship between each PFAS concentration when all other PFAS concentrations are fixed at the median. Adjusted for age, BMI, sex, and ethnicity. The blue line represents the estimate, and the gray area indicates the 95% credible interval.

Figure 3 below displays the univariate exposure-response function, which examines the impact of each PFAS variable on PCSH when the other variables are fixed at the median and all covariates—age, sex, BMI, and ethnicity—are held constant. The results revealed minimal univariate effects, with PFOS showing a slightly positive slope, indicating a potential minimal increase in the risk of PCSH with higher exposure.

PFDE and PFOA show initial dips in both curves which could potentially indicate a threshold effect, where low to moderate exposure levels might have a slight negative impact on the health outcome, but further increases in exposure do not have any more impact.

The curve for PFUA shows a non-linear pattern with a slight dip at moderate exposure levels before rising again at higher exposures with a wide CI, which suggests that PFUA does not have a statistically significant association with PCSH.

#### 3.5.3. Bivariate Exposure-Response Analysis of Joint PFAS Exposures on PCSH

**Figure 4:**
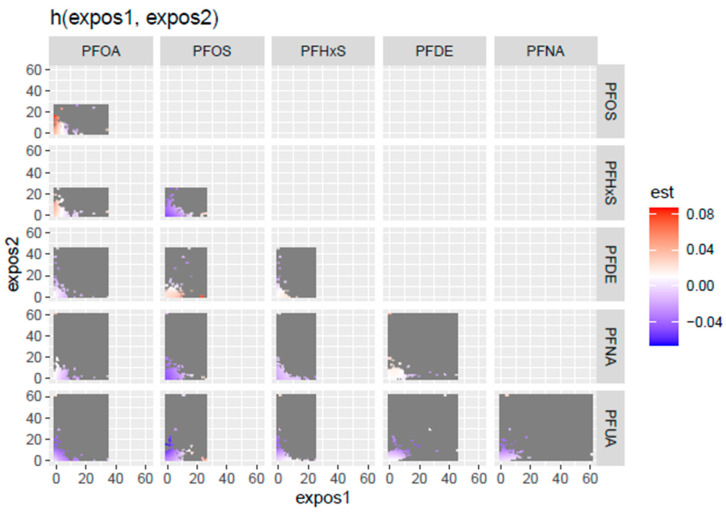
Bivariate exposure-response function, examining the joint effect of PFASs on PCSH. Adjusted for age, BMI, sex, and ethnicity.

Figure 4 shows the bi-variate exposure-response function, examining the joint effects of PFASs on PCSH. The assessment involved investigating the effect of two PFAS concentrations while all others are fixed at a specific percentile and covariates are constant. The color scale (est) shows the estimated effect on PCSH—red indicates a higher positive effect, blue shows a negative effect, and white or grey indicates no effect. The results revealed that the most positive relationship was between PFOA and PFOS at low levels of both concentrations, which suggests a possible synergistic effect on PCSH risk.

**Figure 5:**
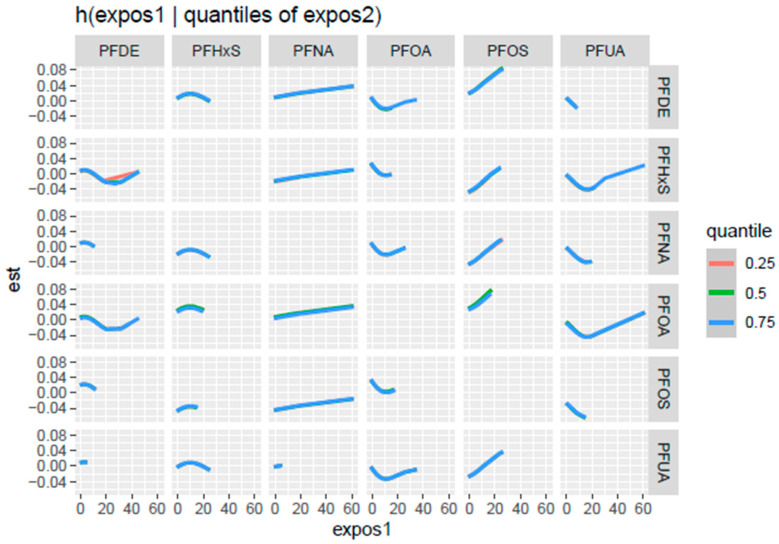
Bivariate exposure-response function examining the relationship between a pair of PFAS concentrations with the second PFASs at the 0.25, 0.5, and 0.75 quantiles, while others are fixed at the median. Adjusted for age, BMI, sex, and ethnicity.

The bivariate exposure-response was further explored (Figure 5) by examining the relationship between specific pairs of PFAS variables and PCSH by fixing the second concentration at 0.25 (red line), 0.5 (green line), and 0.75 (blue line) quantiles while keeping the other PFAS concentration at the median, and the covariates of interest—age, gender, BMI, and ethnicity—were held constant. Each plot cell highlights the relationship between a PFAS variable “expos1” at different concentrations and the estimated effects on PCSH when the second PFAS variable “expos 2” is fixed at the 0.25, 0.5, and 0.75 quantiles represented by the different colored lines, respectively.

As depicted in Figure 5, PFOS (as expos 1) shows a consistent positive association with PCSH, revealing increasing interactions with PFDE, PFHxS, PFNA, and PFUA at the 0.75 quantile, with PFOA at the 0.5 and 0.75 quantiles.

#### 3.5.4. Overall Exposure-Response Analysis of PFASs on PCSH

**Figure 6:**
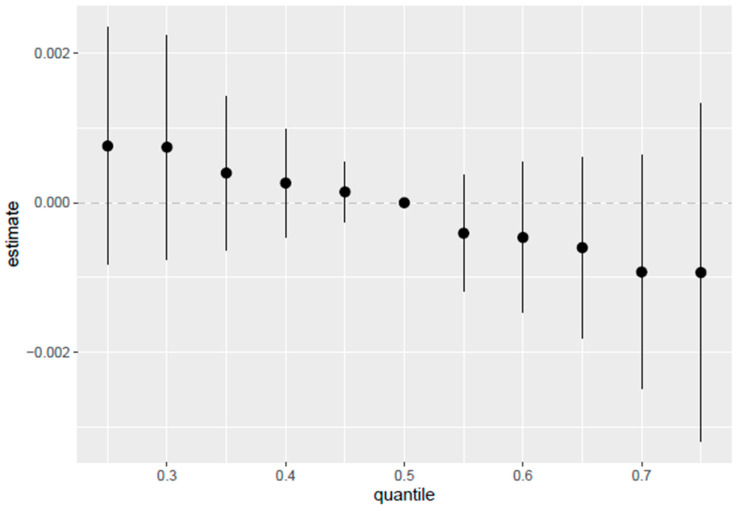
Overall exposure effect and 95% credible interval, examining the effect of all PFAS exposures together on PCSH. The results compare each exposure at quantiles from 0.25 to 0.75 as compared to the median of 0.5. Adjusted for age, BMI, sex, and ethnicity.

The overall exposure-response function (Figure 6), examining the effect of all the PFAS exposures on PCSH fixing all PFAS exposures at different quantiles from 0.25 to 0.75 and the median (0.5 quantile), was used to compare the results from all exposure points. The y-axis represents the estimated effect on PCSH, while the x-axis shows the quantiles of exposure for the exposure variables. Each point in the plot represents the effect estimate at a specific quantile, with the vertical lines indicating the 95% credible intervals around these estimates. The results from the plot reveal that higher risks of PCSH are observed at the lower quantiles of exposures.

#### 3.5.5. Single-Variable Effects of PFASs on PCSH

**Figure 7:**
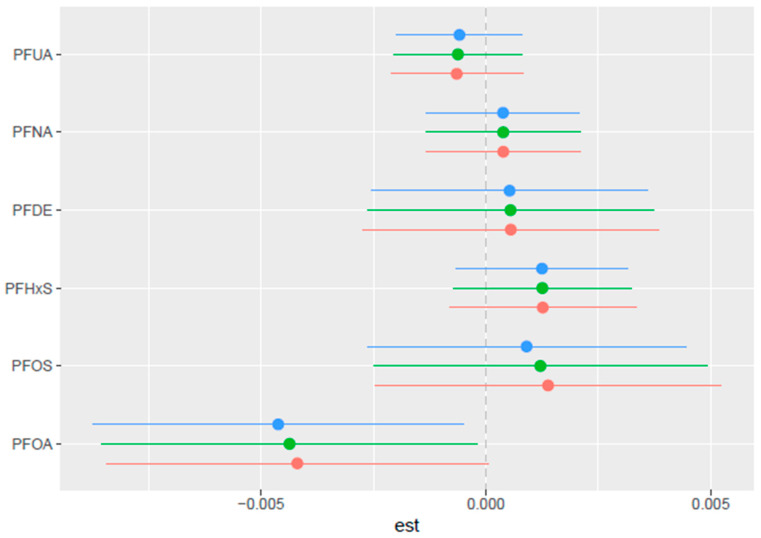
Single-variable effect with 95% credible interval, examining which PFAS has the largest single-variable effect and providing insight into their interaction. Adjusted for age, BMI, sex, and ethnicity. The Blue line indicates the 0.75 quantile, green, the 0.5 quantile, and red, the 0.25 quantile.

The single-variable effect, as shown in Figure 7, assesses the impact of each PFAS variable on PCSH. Figure 7 illustrates the estimated changes in PCSH when a particular exposure shifts from its 0.25 to its 0.75 quantiles, with all other exposures held constant at different quantiles (0.25, 0.50, or 0.75). Blue indicates the 0.75 quantile, green, the 0.5 quantile, and red, the 0.25 quantile. The plot gives an insight into which PFAS has the largest single variable effect and also gives an insight into the interaction of each PFAS with combined exposure to all the other PFASs, providing a comparison of the varying effects of each exposure variable on PCSH, based on the quantiles at which other exposures are held.

#### 3.5.6. Single-Variable Interactions of PFASs on PCSH

**Figure 8:**
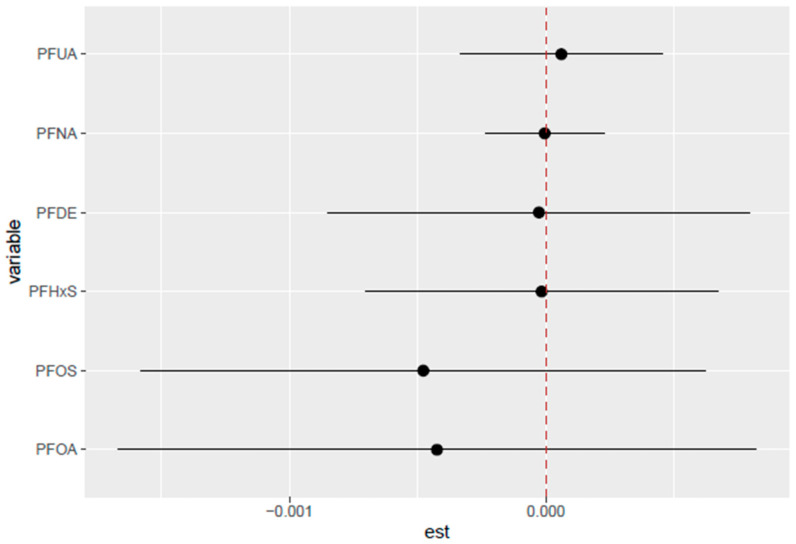
Single-variable interaction with 95% credible interval comparing the effect of each PFAS (from its 0.25 to 0.75 quantile) when all other PFASs are moved from 0.25 to the 0.75 quantile. Adjusted for age, BMI, sex, and ethnicity. Red line indicates no interaction.

Figure 8 highlights the single-variable interaction between PFAS exposures and their association with PCSH, with each horizontal line representing a 95% credible interval for the corresponding variable. The point estimates (black dots) indicate the magnitude and direction of each exposure’s association with PCSH, while the vertical red dotted line at zero represents the null effect. PFOA and PFOS show the most interaction with all other PFASs, as indicated by the deviation of the point estimates from the null effect.

## 4. Discussion

This comprehensive study examined the association between exposure to multiple PFASs and PCSH, using data from the NHANES dataset spanning the period from 1999 to 2012. Our findings reveal a complex relationship between PFAS exposure and PCSH, highlighting the importance of considering non-linear relationships and interactions when evaluating the health impacts of these persistent environmental contaminants.

Initial analyses using *t*-tests indicated a statistically significant difference in PFOS levels between individuals with and without PCSH (*p* = 0.0022), with higher mean PFOS levels being observed in the PCSH group (17.23 ng/mL vs. 13.93 ng/mL). This suggests a potential positive association between PFOS exposure and PCSH risk, which aligns with previous research identifying PFOS as an endocrine disruptor with potential carcinogenic properties [15]. In contrast, traditional regression analyses revealed an inverse association between PFOA and PCSH (β = −0.067, *p* = 0.036; OR = 0.935, 95% CI: 0.876–0.994), suggesting that higher PFOA levels might be associated with decreased odds of PCSH, compared to the results of a previous study [28]. These divergent results underscore a key limitation of conventional regression techniques, which may inadequately capture the complex, nonlinear, or non-monotonic relationships that often characterize environmental exposures [36]. Unlike BKMR, which flexibly models nonlinear dose–response functions and exposure interactions, traditional models assume a linear and additive structure, potentially masking threshold or hormetic effects. It is plausible that PFOA exhibits nonlinearity or effect modification across exposure levels, such as low-dose protective effects and high-dose toxicity, driven by distinct underlying biological mechanisms (e.g., receptor activation, and endocrine disruption thresholds). Consequently, the observed inverse association in the non-BKMR models may not fully reflect the true nature of the relationship, highlighting the importance of mixture-aware and nonlinear approaches such as BKMR for accurate inference in environmental epidemiology.

The study identified significant associations between ethnicity and PCSH (*p* < 0.001), with non-Hispanic Whites being more strongly represented in the PCSH group (57.09%), compared to their proportion in the non-PCSH group (40.04%). Conversely, Mexican Americans were notably underrepresented in the PCSH group (6.03% vs. 19.14% in the non-PCSH group). These findings suggest potential ethnic disparities in the PCSH risk or exposure patterns that warrant further investigation. Male participants comprised a slightly higher proportion of the PCSH group (53.55%) compared to the non-PCSH group (49.44%), although this difference was not statistically significant. These demographic patterns highlight the importance of considering sociodemographic factors when evaluating PFAS-related health risks.

The correlation analyses demonstrated significant relationships among various PFAS compounds, with strong correlations observed between PFOS and PFOA (ϱ = 0.73) and between PFNA and PFDE (ϱ = 0.72), and moderate correlations between PFOS and PFHxS (ϱ = 0.62) and between PFOA and PFHxS (ϱ = 0.58). These findings indicate potential overlapping exposure pathways [37] and highlight the importance of considering PFAS exposures as a mixture of various pollutants rather than as individual exposures [38]. This implies that high correlations in a mixture may indicate similar properties and metabolic processes on hormone-sensitive tissues, which may exceed what can be predicted by individual PFAS concentrations. Clinical investigations in evaluating PFAS exposures will be more effective if assessments consider the cumulative burden of these exposures.

The BKMR analysis provided deeper insights into the complex relationships between PFAS exposure and PCSH. The PIP analysis identified PFOA and PFUA as the most influential predictors for PCSH, with PIPs of 0.1792 and 0.1036, respectively. This finding suggests that these compounds may play a more significant role in PCSH development compared with other PFAS. This implies that PFOA and PFUA may promote greater biological activity in hormone-sensitive tissues compared to the other PFAS concentrations in the study [39,40,41].

Univariate exposure-response functions revealed the limited individual effects of PFASs on PCSH, with PFOS showing a slightly positive slope. However, the bivariate exposure-response analysis uncovered more complex patterns; for example, they revealed non-linear relationships between PFDE, PFHxS, and PFOA. They also revealed an inverse U-relationship between PFHxS and other PFASs. This indicates that at low concentrations of PFHxS, increasing exposures are associated with an increasing risk of PCSH, with the effects reaching a peak at medium concentrations, while further increases in exposure will result in a declining risk of PCSH. Additionally, PFOA had a U-shaped relationship with most other PFAS, highlighting elevated PCSH risk at low and high exposure levels, while PFOS showed a V-shaped relationship with PFHxs and PFOA, indicating high PCSH risk at extreme exposure levels. These results indicate that PFAS concentrations interact differently at varying levels of exposure, and the standard assumption of monotonic dose–response relationships does not hold for these PFAS concentrations. This finding underscores the importance of considering joint exposures when evaluating PFAS-related health risks [38].

Interestingly, the overall exposure-response function indicated higher risks of PCSH at lower quantiles of PFAS exposure. This implies that even at lower concentrations of PFAS exposure, hormonal disruptions of the signaling pathways in the development of cancers associated with sex hormones are likely to occur [42,43]. This pattern differs from traditional dose–response relationships and may indicate complex endocrine-disrupting mechanisms that operate differently at various exposure levels. Lower quantiles being associated with PCSH may suggest that at a certain threshold, the effects may be confounded by other unmeasured factors such as diet, socioeconomic, demographic and lifestyle variations, exposure periods, metabolic differences, etc.

The stronger association between PFAS exposure and PCSH at lower exposure levels (below the 0.5 quantile) suggests that even relatively low PFAS concentrations may impact hormone-sensitive tissues [42]. This finding has important public health implications, as it indicates that current exposure levels in the general population may not be without risk.

In addition, the single-variable interaction analysis suggests that for PFOA, as the levels of the remaining PFASs increase from their 25th to 75th percentiles, the estimated risk of PCSH associated with PFOA also increases. A similar pattern is observed for PFOS. This pattern indicates that both PFOA and PFOS may interact more strongly with other PFASs, potentially exerting a greater combined influence on PCSH compared to the other compounds. PFOA and PFOS possess estrogen-mimicking characteristics and can bind to sex hormone-binding globulin (SHBG), potentially interfering with the synthesis and distribution of sex hormones [18]. This can lead to the alteration of bioavailability or the disruption of the transport of estrogen and testosterone. This mechanism could explain the observed associations with hormone-dependent cancers [44,45,46].

### 4.1. Strengths and Limitations of the Study

This study has several strengths, including its large sample size, the use of advanced statistical methods to capture complex relationships, and the consideration of multiple PFAS compounds. The use of BKMR allowed us to identify non-linear relationships and interactions that might not be apparent with traditional regression methods.

However, some limitations of the study should be acknowledged. First, the cross-sectional nature of the NHANES data limits our ability to establish causality or temporal relationships between PFAS exposure and PCSH. Second, while we adjusted for several important covariates, residual confounding cannot be ruled out. Third, our study relied on self-reported cancer diagnoses, which may be subject to recall bias.

### 4.2. Public Health Implications

Despite these limitations, our findings have important public health implications. The complex relationships between PFAS exposure and PCSH risk highlight the need for a precautionary approach to PFAS regulation and exposure reduction. The potential effects at lower exposure levels suggest that current regulatory standards may not be sufficiently protective, particularly for vulnerable populations.

The differences observed across ethnic groups warrant further investigation and may indicate the need for targeted interventions or monitoring in specific populations. Additionally, the complex interactions among PFAS compounds underscore the importance of considering mixture effects in risk assessment and regulation, rather than focusing solely on individual compounds.

### 4.3. Future Research Directions

Future research should focus on several key areas. Longitudinal studies are needed to better establish the temporal relationship between PFAS exposure and PCSH development. More detailed analyses of exposure timings and duration would provide insights into the critical windows of susceptibility. Studies incorporating the biomarkers of endocrine disruption and hormone levels would help elucidate the mechanisms underlying the observed associations.

Further investigation of the non-monotonic dose–response relationships observed in our study would improve our understanding of the health impacts of PFASs at various exposure levels.

## 5. Conclusions

In conclusion, our study provides evidence of complex relationships between PFAS exposure and pan-cancers associated with sex hormones. While initial findings suggested a positive association between PFOS and PCSH, more advanced analyses revealed non-linear relationships and interactions among PFAS compounds. These findings emphasize the importance of evaluating PFASs as mixtures rather than as individual compounds and using techniques that can capture complex exposure-response patterns.

The potential health impacts of PFASs at current exposure levels in the general population highlight the need for continued monitoring, research, and preventive measures to reduce exposure to these persistent environmental contaminants. As our understanding of these relationships continues to evolve, a precautionary approach to PFAS regulation and exposure reduction is warranted to protect public health.

## Figures and Tables

**Table 1:** Demographic characteristics of the study participants.

Variable	Description	Frequency (*n*)	Percentage (%)
Gender	Male	7117	49.52
	Female	7256	50.48
Race/Ethnicity	Mexican American	2714	18.88
	Other Hispanic	1240	8.63
	Non-Hispanic White	5803	40.37
	Non-Hispanic Black	3233	22.49
	Other races	1383	9.62
Having PCSH	No	14,087	98.04
	Yes	282	1.96

**Table 2:** Mean levels of critical variables in the study.

Variable	Mean (SD)	Minimum	Median (IQR)	Maximum
PFOA	3.31 (2.89)	0.07	2.67 (1.57–4.30)	104.00
PFOS	13.99 (16.02)	0.14	9.80 (4.80–18.00)	435.00
PFHxS	2.31 (3.19)	0.07	1.50 (0.80–2.60)	82.00
PFDE	0.34 (0.55)	0.07	0.20 (0.14–0.400	25.20
PFNA	1.15 (1.30)	0.06	0.90 (0.56–1.39)	80.77
PFUA	0.24 (0.46)	0.07	0.14 (0.10–0.20)	28.50

**Table 3:** Statistical analysis (*t*-test) of the association between PFAS exposures among participants with PCSH and without PCSH.

Variable	Mean (SD) *n* = 14,373	PCSH (+) *n* = 282	No PCSH (−) *n* = 14,087	*p*-Value
PFOA	3.31 (2.89)	3.32 (2.40)	3.31 (2.90)	0.9216
PFOS	13.99 (16.02)	17.23 (17.77)	13.93 (15.96)	0.0022 **
PFHxS	2.31 (3.19)	2.47 (2.45)	2.31 (3.20)	0.2865
PFDE	0.34 (0.55)	0.35 (0.34)	0.34 (0.55)	0.7333
PFNA	1.15 (1.30)	1.24 (1.05)	1.15 (1.30)	0.1643
PFUA	0.24 (0.46)	0.24 (0.27)	0.24 (0.46)	0.9506

** Indicates statistical significance, *p* < 0.05.

**Table 4:** Statistical analysis (chi-squared test) of the differences between groups of participants with PCSH and without PCS.

Variable	Description	PCSH (+) *n* (%)	No PCSH (−) *n* (%)
Gender	Male	151 (53.55)	6965 (49.44)
	Female	131 (46.45)	7122 (50.56)
Race/Ethnicity	Mexican American	17 (6.03)	2696 (19.14)
	Other Hispanic	21 (7.45)	1219 (8.65)
	Non-Hispanic White	161 (57.09)	5641 (40.04)
	Non-Hispanic Black	59 (20.92)	3172 (22.52)
	Other races, including multiracial	24 (8.51)	1359 (9.65)

**Table 5:** Linear regression analysis, showing the association between the predictor variables and PCSH.

	Coefficients	Std. Error	Z Value	*p*-Value
PFOA	−0.067	0.032	−2.096	0.036 **
PFOS	−0.0004	0.005	−0.090	0.928
PFHxS	0.011	0.024	0.443	0.657
PFDE	0.088	0.198	0.441	0.658
PFNA	0.034	0.033	1.021	0.307
PFUA	−0.283	0.2978	−0.950	0.342

** Indicates statistical significance, *p* < 0.05.

**Table 6:** Logistic regression analysis, showing the association between the predictor variables and PCSH.

Variables	Odds Ratio	2.5%	97.5%
PFOA	0.935	0.876	0.994
PFOS	1.000	0.989	1.009
PFHxS	1.011	0.959	1.055
PFDE	1.092	0.681	1.443
PFNA	1.034	0.935	1.093
PFUA	0.754	0.405	1.272

**Table 7:** Posterior inclusion probabilities (PIP) of the influence of PFOA, PFOS, PFHxS, PFDE, PFNA, and PFUA on pan-cancers associated with sex hormones.

Variable	PIP
PFOA	0.1792
PFOS	0.0772
PFHxS	0.0404
PFDE	0.0690
PFNA	0.0032
PFUA	0.1036

## Data Availability

The NHANES dataset is publicly available online, accessible at https://www.cdc.gov/nchs/nhanes/ (accessed on 4 April 2025).
